# Nivolumab as Second-Line Therapy Improves Survival in Patients with Unresectable Hepatocellular Carcinoma

**DOI:** 10.3390/cancers16122196

**Published:** 2024-06-11

**Authors:** Faisal M. Sanai, Hassan O. Odah, Kanan Alshammari, Adnan Alzanbagi, Murooj Alsubhi, Hani Tamim, Ashwaq Alolayan, Ahmed Alshehri, Saleh A. Alqahtani

**Affiliations:** 1 Gastroenterology Section, Department of Medicine, King Abdulaziz Medical City, Ministry of the National Guard-Health Affairs, Jeddah 21423, Saudi Arabia; hassan.odah@drsulaimanalhabib.com; 2 King Abdullah International Medical Research Center, Jeddah 22384, Saudi Arabia; 3 King Saud bin Abdulaziz University for Health Sciences, Jeddah 22384, Saudi Arabia; 4 Oncology Department, King Abdulaziz Medical City, King Abdullah International Medical Research Center, Ministry of National Guard Health Affairs, Riyadh 11481, Saudi Arabiaalolayanas@ngha.med.sa (A.A.); 5 Gastroenterology and Hepatology Department, King Abdullah Medical City, Makkah 57657, Saudi Arabia; alzanbagi.a2@kamc.med.sa; 6 Department of Medical Imaging, King Abdulaziz Medical City, Riyadh 11426, Saudi Arabia; alsubhimo2@mngha.med.sa; 7 College of Medicine, Alfaisal University, Riyadh 11533, Saudi Arabia; htamim@aub.edu.lb; 8 Department of Internal Medicine, American University of Beirut Medical Center, Beirut 1107, Lebanon; 9 Adult Medical Oncology Section, Adult Oncology Department, Princess Noorah Oncology Center, King Abdulaziz Medical City, Jeddah 22384, Saudi Arabia; a.s.a.alshehri@gmail.com; 10 Organ Transplant Centre of Excellence, King Faisal Specialist Hospital & Research Centre, Riyadh 11564, Saudi Arabia; salqaht1@jhmi.edu; 11 Division of Gastroenterology and Hepatology, Johns Hopkins University, Baltimore, MD 21218, USA

**Keywords:** hepatocellular carcinoma, Barcelona Clinic Liver Cancer, systemic therapy, treatment, sorafenib, nivolumab, survival

## Abstract

**Simple Summary:**

The current options for second-line therapy in unresectable liver cancer are limited, with an evolving role for immunotherapeutic regimens. This study examines the role of nivolumab as a second-line treatment option in 42 patients with liver cancer who had failed first-line therapy with sorafenib and compared outcomes in another 38 patients who were maintained on sorafenib therapy. Our results show that nivolumab prolongs survival in such patients compared to those who were continued on sorafenib therapy. This study adds to the growing body of research that nivolumab may be an effective treatment option in patients with unresectable and advanced liver cancer.

**Abstract:**

Background: Limited data exists for the efficacy and outcomes of nivolumab as a second-line treatment for unresectable hepatocellular carcinoma (uHCC). We aimed to assess the efficacy and safety of nivolumab in patients with uHCC who experienced disease progression during sorafenib treatment. Methods: In this retrospective, observational, multicenter study, adult Child-Turcotte-Pugh A/7B patients with uHCC who tolerated sorafenib therapy but showed disease progression switched to second-line intravenous nivolumab (*n* = 42). A similar number of consecutive, unselected patients who were maintained on sorafenib therapy, regardless of tumoral response or progression, served as historical controls (*n* = 38). The primary endpoint was overall survival (OS, defined as the time from starting sorafenib in either group up to death due to any cause) and analyzed by intention-to-treat. Results: The mean age of the overall cohort was 72.4 ± 10.1 years, of whom 87.5% were males and 58.8% had underlying viral etiology. Patients in the two cohorts were similar, except those who received nivolumab had more co-morbidities (70.0% vs. 15.4%), ECOG-2 status (21.4% vs. 15.8%), BCLC stage C (81.0% vs. 47.4%), and extravascular invasion (54.4% vs. 21.8%) (*p* < 0.05 for all). More patients in the nivolumab arm were Child-Turcotte-Pugh B (35.7% vs. 21.1%, *p* = 0.15). Median OS was 22.2 months (95% CI: 8.9–49.8) on second-line nivolumab and 11.0 months (95% CI: 3.6–18.4) on sorafenib alone (HR 1.93; 95% CI: 1.1–3.3, *p* = 0.014). Median OS after starting nivolumab was 10.2 months, and time-to-progression was 4.9 months (95% CI: 3.2–6.3). Conclusion: Nivolumab is an effective second-line treatment option in patients with uHCC who progress on sorafenib, with significantly improved OS. These early real-life data offer encouraging results, similar to those shown in Phase I/IIa clinical trials. Further investigations are warranted for the use of nivolumab as a monotherapy.

## 1. Introduction

Hepatocellular carcinoma (HCC) represents the third most frequent cause of cancer-related death in men and the sixth most prevalent malignancy overall [1]. Many patients are diagnosed at advanced stages of HCC, and disease recurrence or progression after initial treatment is common [2,3]. Sorafenib, an oral multikinase inhibitor, which improved overall survival (OS) compared to a placebo in the SHARP trial [4], has been the only viable treatment for unresectable HCC (uHCC) over the past decade. However, recent successful phase II/III trials of first- or second-line therapies have expanded the treatment landscape for patients with advanced HCC [5,6,7].

Despite the rapid expansion of treatment options, achieving long-term survival in patients with uHCC is limited by the occurrence of therapeutic resistance [8]. The significant role of innate and adaptive immunity in influencing the pathogenesis and progression of HCC has made it an appealing target for antibody therapy against immune co-inhibitory signals in the tumor microenvironment [9,10,11]. Monoclonal antibodies against programmed cell death protein 1 (PD-1) or its ligand 1 (PD-L1) have recently become standard-of-care for some cancers, such as non-small cell lung cancer, melanoma, and others [12]. In HCC, the expression of PD-1/PD-L1 is associated with an increased risk of recurrence and shortened survival rates [13], suggesting a rationale for inhibition [9]. Nivolumab, an immune checkpoint inhibitor that blocks PD-1, has shown durable responses and prolonged long-term survival in the CheckMate 040 trial, a single-arm, open-label phase I/II study [5]. It became the first PD-1 inhibitor to receive conditional approval by the US Food and Drug Administration (FDA) in 2017 for treating uHCC in Child-Turcotte-Pugh (CTP) class ‘A’ patients after prior tyrosine kinase inhibitor (TKI) exposure. Subsequently, in the CheckMate 459 phase III study of nivolumab versus sorafenib as first-line treatments in advanced HCC, nivolumab appears to benefit a proportion of patients, especially in the context of a radiological response, despite that statistical significance was not achieved for the primary endpoint of OS [14].

A few retrospective cohort studies and case series have reported on the safety and effectiveness of immune checkpoint inhibitors for advanced HCC patients in first- [15,16,17] and second-line therapy, albeit with a small number of patients. In this study, we aim to report real-world data on the clinical outcomes and safety of nivolumab in a retrospective cohort of patients with uHCC in a second-line setting, after previous failure with sorafenib.

## 2. Patients and Methods

### 2.1. Study Design and Participants

In this multicenter, retrospective, observational study, we evaluated the efficacy and safety of nivolumab as a second-line therapy in patients with uHCC who tolerated but experienced disease progression during sorafenib treatment. Between January 2012 and March 2023, consecutive, unselected patients with uHCC who tolerated sorafenib therapy were included as a historical cohort of controls. Of these, after April 2018, patients who showed disease progression and consented were prescribed nivolumab as a second-line therapy (Supplementary Figure S1). Patients were included from three centers across Saudi Arabia: King Abdulaziz Medical City—Jeddah, King Abdallah Medical City—Makkah, and King Abdulaziz Medical City—Riyadh from Apr 2018 to March 2023. Patient data were collected from the Saudi Observatory Liver Disease (SOLID) registry, which is a prospective, multicenter observational registry. The study was conducted in accordance with the guidelines of the Declaration of Helsinki and the principles of Good Clinical Practice, and the institutional review boards of the participating centers approved the study.

Eligible patients were at least 18 years of age with radiologically or histologically confirmed HCC and had a life expectancy of at least 12 weeks, received at least one dose of the nivolumab, and were considered suitable for systemic therapy, as their disease was not amenable to curative or loco-regional therapy. Patients were also required to have CTP scores of 7 or less (CTP class A or 7B), either BCLC stage B or C, at least one tumor lesion that could be accurately measured in at least one dimension (according to RECIST 1.1 [Response Evaluation Criteria in Solid Tumors]), and an Eastern Cooperative Oncology Group (ECOG) performance status of 0, 1, or 2. Patients with advanced hepatic decompensation (CTP ≥ 8) or BCLC stage 0/A or D, or who had received nivolumab as a first-line or third-line systemic therapy were excluded (Supplementary Figure S1). Patients with previous or concurrent cancer that is distinct from HCC in primary site or histology, or that is curatively treated >3 years prior to study entry, and with renal failure requiring hemo- or peritoneal dialysis, high-grade portosystemic encephalopathy (PSE) or ascites, liver transplantation, autoimmune liver disease, or human immunodeficiency virus infection were also excluded.

### 2.2. Treatment Administration and Outcome Measures

Patients who tolerated 400–800 mg/day of sorafenib therapy but had progressing HCC discontinued sorafenib treatment and were administered second-line nivolumab intravenously at a dose of 3 mg/kg of body weight every 2 weeks. Dose modifications were made based on toxicity in accordance with the summary of product characteristics for nivolumab and sorafenib. Treatment was continued until unacceptable toxicity occurred in the sorafenib-only group and/or until disease progression in the sorafenib-nivolumab sequence group. Patient demographics and clinical data were collected retrospectively and were prospectively curated and updated at each participating center. Patients were assessed in terms of ECOG performance status, macrovascular invasion, extrahepatic disease, and α-fetoprotein level to assess the best supportive care plus oral sorafenib or intravenous nivolumab as a second-line therapy.

The primary endpoint was OS (defined as the time from starting sorafenib in either group up to death due to any cause) and analyzed by the intention-to-treat population. Time-to-tumor progression (TTP) was defined as the time from the date of the first dose of sorafenib in either group to the date of death or the date of radiological evidence of tumor progression while on sorafenib. Tumor assessment was carried out using dynamic computerized tomography (CT) or magnetic resonance imaging (MRI) every 8–12 weeks according to the Response Evaluation Criteria in Solid Tumors (RECIST) version 1.1.

### 2.3. Safety Analysis Endpoints

Patients were followed up for safety at every cycle or clinic visit. The National Cancer Institute Common Terminology Criteria for Adverse Events version 5.0 was used to grade side effects at every contact with the patient and based on a review of medical records, laboratory findings, or imaging results. The attribution of causality to sorafenib or nivolumab was based on the assessment of the treating physician. Safety was assessed between the first dose and up to 90 days after the last dose of sorafenib or nivolumab. Safety outcomes included all-cause- and treatment-related adverse events (TRAEs), immune-related AEs, or those requiring steroids, hospitalization, or resulting in the discontinuation of treatment.

### 2.4. Statistical Analysis

Demographic data were summarized using descriptive statistics, mainly numbers and percentages for nominal data, whereas continuous variables were expressed as mean ± standard deviation (SD) or median and interquartile range (IQR). Nominal data were compared using Fisher’s exact test or the chi-square test, as appropriate. On the other hand, continuous variables were compared using the independent *t*-test or the Mann-Whitney *U* test, as appropriate. OS curves in the sorafenib and nivolumab groups, and in the patient subgroups, were calculated using the Kaplan–Meier method, and these were compared with the log-rank test. The hazard ratios (HRs) for survival outcomes and their 95% confidence intervals (CIs) were calculated. SPSS (IBM SPSS 28.0, Armonk, NY, USA) was used to perform all statistical analyses. A two-sided *p*-value of <0.05 was considered statistically significant.

## 3. Results

### 3.1. Baseline Characteristics of Patients

Of the overall included patients in the study, 42 were prescribed nivolumab as a second-line therapy, while the rest (*n* = 38) received nivolumab only. Patient demographics and baseline disease characteristics are shown in Table 1. The mean age of the overall cohort was 72.4 ± 10.1 years, 70 (87.5%) were males, and 47 (58.8%) had underlying viral etiology. Of the included patients (*n* = 80), 52 (65.0%) had BCLC stage C, 23 (28.8%) had CTP 7B disease, and 15 (18.8%) had ECOG 2; co-morbidities included diabetes mellitus (DM) in 24 (30.0%), hypertension in 24 (30.0%), and chronic kidney disease (CKD) in 5 (6.3%) patients. Portal vein thrombosis (PVT) existed in 36 (45.0%), metastases in 31 (38.8%), and hepatic decompensation existed in 14 (17.5%) patients, including variceal bleeding in 5 (6.3%), ascites in 12 (15.0%) and PSE in 3 (3.8%). Fourteen patients (17.5%) had a histological evaluation of the liver tumor, of whom 12 (85.7%) had the degree of differentiation mentioned (well differentiated, *n* = 4 [25%], moderately differentiated, *n* = 7 [58.3%], poorly differentiated, *n* = 1 [8.3%]). Biopsy was performed for disease diagnosis at baseline, while none was performed during disease evolution.

Patients in the nivolumab arm were younger (70.0 ± 9.8 vs. 75.0 ± 9.8 years), had more co-morbidities (70.0% vs. 15.4%), ECOG 2 status (21.4% vs. 15.8%), BCLC stage C (81.0% vs. 47.4%), and extravascular invasion (54.8% vs. 21.1%) (*p* < 0.05 for all). Moreover, patients who received nivolumab were more likely to have higher CTP status than the sorafenib-only group (CTP 7B; 35.7% vs. 21.1%), although this did not reach statistical significance (*p* = 0.15; Table 1). In the subcategory of patients who were CTP A, the nivolumab-treatment arm had more co-morbidities, BCLC stage C (81.5% vs. 50.0%), and more metastatic disease (63.0% vs. 21.3%) (*p* < 0.05 for all). Other characteristics were not significantly different between the two arms (Table 2). Additionally, no differences were observed in baseline characteristics of the subcategory of BCLC stage B patients between the two treatment arms (Table 3).

### 3.2. Treatment Outcomes and Survival

**Figure 1:**
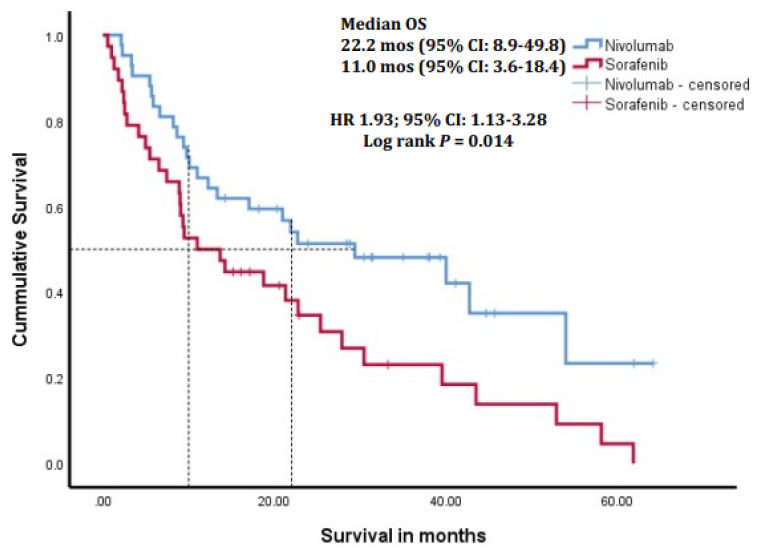
Kaplan–Meier survival curves of patients receiving sorafenib only, or sorafenib followed by second-line nivolumab treatment, showing a significantly better median overall survival (*p* = 0.014) in the patients receiving nivolumab. OS, overall survival; CI, confidence interval; HR, hazard ratio.

At the data cutoff of 1 April 2023, the median follow-up duration in the overall cohort was 23.4 months (IQR, 12.1–46.5), 33.1 months (IQR, 11.8–50.3) in the second-line nivolumab arm, and 19.2 months (IQR, 12.0–33.7) in the sorafenib-only arm. Patients who received nivolumab after disease progression on sorafenib had a significantly longer median OS (mOS) of 22.2 months (95% CI: 8.9–49.8) compared to 11.0 months (95% CI: 3.6–18.4) in patients who received sorafenib only (HR 1.93; 95% CI: 1.13–3.28, *p* = 0.014; Figure 1). The mOS after starting nivolumab was 10.2 months. There was no difference in the tumor response rates between the two treatment arms based on the investigator-assessed RECIST1.1 (Table 4). The median TTP from the start of sorafenib to the progression of sorafenib in the nivolumab arm was 4.9 months (95% CI: 3.2–6.3) and 5.2 months (95% CI: 3.9–8.8) in the sorafenib-only arm (HR 0.60; 95% CI: 0.33–1.11, *p* = 0.201; Supplementary Figure S2).

In the overall study population, the median follow-up duration of the CTP A patients was 21.1 months (IQR, 12.1–44.6) and 30.4 months (IQR, 9.9–63.9) for CTP B patients. CTP A patients had a longer mOS (22.7 months; 95% CI: 10.9–34.5) compared to CTP B patients (10.1 months; 95% CI: 5.5–14.7) but this did not reach statistical significance (*p* = 0.481; Supplementary Figure S3A). The median follow-up duration of the BCLC stage B patients was 19.2 months (IQR, 10.5–45.5), and the same was 26.2 months (IQR, 12.1–46.6) for BCLC C patients. Similarly, there was no difference in mOS between BCLC stage B (21.3 months; 95% CI: 1.1–41.5) and BCLC C patients (18.7 months; 95% CI: 9.2–28.3, *p* = 0.76) (Supplementary Figure S3B).

**Figure 2:**
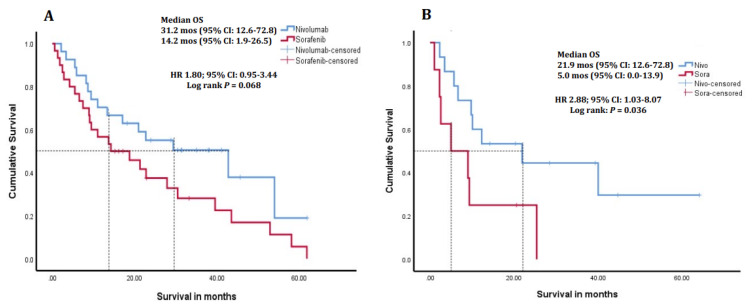
Kaplan–Meier survival curves of patients receiving sorafenib only or sorafenib followed by second-line nivolumab treatment in sub-groups of (**A**) Child-Turcotte-Pugh class A, and in (**B**) Child-Turcotte-Pugh class B. Child-Turcotte-Pugh A patients receiving nivolumab had a trend toward longer median OS, although this did not reach statistical significance (*p* = 0.068). Child-Turcotte-Pugh B patients receiving nivolumab had a longer median OS (*p* = 0.036) compared to those who only received sorafenib. OS, overall survival; CI, confidence interval; HR, hazard ratio.

**Figure 3:**
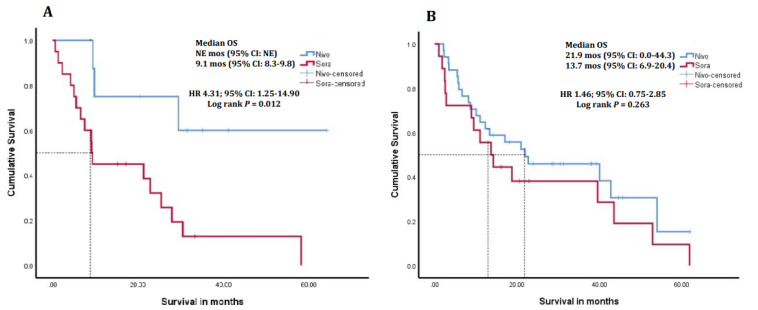
Kaplan–Meier survival curves of patients receiving sorafenib only, or sorafenib followed by second-line nivolumab treatment in sub-groups of (**A**) BCLC B and (**B**) BCLC C. BCLC B patients who received nivolumab had a longer median overall survival (*p* = 0.012) that had not reached a median time point by study closure. BCLC C patients who received nivolumab, or those who received sorafenib only had a similar median overall survival (*p* = 0.263). OS, overall survival; NE, non-estimable; CI, confidence interval; HR, hazard ratio; BCLC, Barcelona Clinic Liver Cancer.

In CTP class A patients, the median follow-up duration was 32.4 months (IQR, 12.1–46.8) and 17.5 months (IQR, 12.0–32.9) for the nivolumab and sorafenib-only arms, respectively. In CTP class B patients, the median follow-up duration was 33.7 months (IQR, 9.3–63.9) and 24.9 months (IQR, 12.4–93.7) for the nivolumab and sorafenib-only arms, respectively. In BCLC B patients, the median follow-up duration was 45.9 months (IQR, 10.2–64.2) and 18.2 months (IQR, 11.1–31.3) for the nivolumab and sorafenib-only arms, respectively. CTP A patients in the nivolumab arm showed a trend to longer mOS, with 31.2 months (95% CI: 12.6–72.8) compared to 14.2 months (95% CI: 1.9–26.5) in the sorafenib only arm (HR 1.80; 95% CI: 0.95–3.44, *p* = 0.068; Figure 2A). CTP B patients who received nivolumab had a significantly longer mOS of 21.9 months (95% CI: 12.6–72.8) compared to 5.0 months (95% CI: 12.6–72.8) in those treated with sorafenib only (HR 2.88; 95% CI: 1.03–8.07, *p* = 0.036) (Figure 2B). Additionally, mOS in patients with BCLC stage B disease at baseline was not estimable with nivolumab, and was 9.1 months (95% CI: 8.3–9.8) with sorafenib (HR 4.31; 95% CI: 1.25–14.90, *p* = 0.012), while in BCLC stage C patients was 21.9 months (95% CI: 0–44.3) and 13.7 months (95% CI: 6.9–20.4), respectively (HR 1.46; 95% CI: 0.75–2.85, *p* = 0.263; Figure 3A,B).

### 3.3. Safety

The median duration of treatment with sorafenib was 5.2 months (IQR, 1.5–7.6) within the sorafenib-only group and 3.1 months (IQR, 2.2–6.3) within the nivolumab group. Patients treated with nivolumab received a mean of 6.5 cycles of therapy. The overall incidence of TRAEs for patients treated in the sorafenib-only and nivolumab arms was 44.7% and 35.7%, respectively (*p* = 0.16). In both arms, the most common adverse events were skin rash, gastrointestinal symptoms, and increased aminotransferase levels (Supplementary Table S1). No significant increase in the risk of grade 3/4 TRAEs or deaths related to the medications was observed in both arms. One patient on nivolumab experienced bronchospasm with lung infiltrates that were treated with bronchodilators, steroids, and antibiotics, and required a delay in the treatment cycle. Systemic steroids were administered to 5 (13.2%) and 8 (19.0%) patients in the sorafenib-only and nivolumab arms, respectively. Hepatic decompensation events were similar in the nivolumab- (*n* = 21, 50.0%) and sorafenib-treated (*n* = 21, 55.3%) arms.

## 4. Discussion

In this study, we retrospectively compared the efficacy and safety of nivolumab as a second-line therapy in patients with uHCC who experienced disease progression on sorafenib treatment compared to patients who maintained only sorafenib treatment. The median OS was significantly longer in patients treated with nivolumab (22.2 months) versus those treated with sorafenib only (11.0 months). These findings are in line with the CheckMate 040 trial that showed a mOS of 15.1 months with nivolumab as a second-line option after treatment failure with sorafenib. However, limited real-world experience exists with nivolumab, particularly as a second-line option. Lee et al. reported a survival of 5.4 months with nivolumab in sorafenib-experienced BCLC C patients [18]. Similar results were demonstrated by Fessas et al., who showed a survival of 12.2 months when nivolumab was administered as either a first- or second-line therapy in a cohort of mostly BCLC stage B/C patients [19].

In our cohort, nivolumab showed a longer OS compared to sorafenib, despite the presence of multiple negative confounders, such as the presence of more co-morbidities, more patients with ECOG 2 status, BCLC C status, extravascular invasion, and CTP B status in the nivolumab arm. In previous analyses of patients with uHCC, the CTP score, BCLC stage, and performance status have been prognostically valuable in predicting OS [20,21,22,23]. The survival of sorafenib-treated CTP B patients in our study (5 months) was comparable to real-life data reported from the GIDEON registry (5.2 months) [20].

Similar to this analysis, several studies have evaluated the safety and efficacy of second-line systemic therapy in patients with uHCC. In the RESORCE trial, post hoc analysis of regorafenib as a second-line therapy showed a mOS of 26.0 months from the start of sorafenib until death [24]. However, the regorafenib-treated patients were almost exclusively CTP A (98%), while the nivolumab cohort in our study had a substantially higher number of CTP B patients (35.7%). More recently, lenvatinib, as a second-line option after atezolizumab/bevacizumab failure, showed a longer survival in CTP A patients (not reached) compared to the overall group (15.7 months) [25]. In another retrospective study, no significant differences regarding survival were found between CTP class B patients given atezolizumab/bevacizumab (5.8 months) or lenvatinib (8.8 months) as an initial systemic treatment for uHCC [26]. In the CTP B cohort of the CheckMate 040 trial, the survival outcome was considerably lower (7.6 months) than was seen in our patients [5]. In another study by Choi et al., nivolumab treatment resulted in lower survival in CTP B patients (10.0 vs. 2.6 months) [17]. In our nivolumab-treated cohort, although CTP A patients had a longer survival (31.2 months), CTP B patients also experienced a clinically relevant prolonged survival of 21.9 months, substantially more than other systemic therapies, and previous reports of nivolumab in CTP B patients. This is possibly related to the inclusion of higher CTP scores (B8–9) in the other cohorts [5,17], whereas our cohort principally included only CTP B7 patients. Such differences in survival related to CTP class could potentially be due, in part, to some patients’ impaired liver status not having enough time to maintain nivolumab treatment due to progressive liver dysfunction. Indeed, Choi et al. observed that 22.5% of CTP B patients discontinued treatment due to death, mostly resulting from liver function deterioration, whereas only 5.3% of CTP A patients ceased the treatment due to death [17].

The relevance of maintained hepatic reserve appears crucial in prolonging survival in uHCC [21,22,25]. Checkpoint antibody inhibitors such as nivolumab and atezolizumab/bevacizumab have been able to maintain liver function for prolonged periods [27], possibly due to high specificity and affinity of monoclonal antibodies, resulting in low off-target effects. On the other hand, TKIs, such as sorafenib, are known to have off-target effects [28], thereby contributing to the loss of hepatic reserve and development of AEs. 

Few studies have evaluated the role of systemic therapies in BCLC stage B disease, particularly in the context of second-line treatments. In an exploratory analysis of the IMbrave150 study, survival in patients with BCLC stage B disease was 25.8 months with atezolizumab/bevacizumab and 21.9 months with sorafenib [29]. In the BCLC stage B subgroup of the REFLECT trial, survival with lenvatinib was 18.5 months vs. 17.3 months with sorafenib [6]. On the other hand, in the CheckMate 459 trial, first-line nivolumab in the BCLC stage B subgroup showed a trend toward reduced survival with nivolumab vs. sorafenib (HR: 1.35; 95% CI: 0.86–2.11) [14]. Nonetheless, in our cohort of BCLC B patients, nivolumab treatment resulted in a longer mOS (not reached after a median follow up of 45.9 months) than the sorafenib-only group (9.1 months, *p* = 0.012). These data suggest that nivolumab may aid in delaying or preventing disease progression when administered in patients at an earlier stage of the disease, thus contributing to the growing body of evidence that advocates for the early uptake of systemic therapies in BCLC stage B disease unsuitable for or unresponsive to transarterial chemoembolization.

Ascertaining the clinical value of PD-1 monotherapy has been challenging in clinical studies because of the lack of consistent responses and predictive markers of response. The CheckMate 040 collected tumoral tissue at baseline for the analysis of PD-L1 expression and showed that clinically meaningful objective responses occurred regardless of tumor PD-L1 expression [5]. As such, as in our study where only 17.5% of the patients had undergone liver tumoral biopsy, real-life experiences of HCC treatment generally do not utilize tumoral targets in guiding systemic therapy due to a lack of perceived clinical benefit. However, more recently, a systematic review and meta-analysis in 1330 HCC patients treated with PD-1/PD-L1 inhibitors showed that positive PD-L1 expression is associated with better overall response rates in advanced HCC patients [30]. These findings suggest that assessing tumor biology, including tumoral biomarker expression, may become crucial in the future as the treatment landscape with systemic therapies expands.

Both drugs were well tolerated during the treatment period. The safety and tolerability profile of nivolumab in this analysis was consistent with the known safety profile of the drug and with the underlying disease. No new or unexpected SAEs were identified for nivolumab in this study. The occurrence of TRAEs across all categories was similar to the nivolumab and sorafenib groups. One patient experienced bronchospasm requiring bronchodilators, steroids, and antibiotics. While this study did not record the quality of life (QoL) in either group of patients, this has been assessed systematically in the CheckMate 040 trial with multiple questionnaires and demonstrated consistently stable findings with no significant change from the baseline [5]. Ultimately, the need to evaluate the detailed impact of these systemic therapies (i.e., sorafenib vs. nivolumab) on QoL domains remains a crucial area of research that needs to be addressed in the complex treatment landscape of HCC [6].

Our study is limited by several factors that are inherent to retrospective analyses, such as inclusion bias, challenges in data collection, and confounding. Considering that most clinical trials only include patients with CTP class A to avoid competing risks of death from cirrhosis on the overall outcome, this retrospective cohort study may provide valuable information for evaluating the effectiveness and safety of nivolumab in a real-life setting where the patients tend to be more heterogeneous than those in clinical trials. Second, the limited sample size for both treatment arms in this analysis prevented meaningful interpretation of results, evidenced by the large confidence intervals in all analyses. Results from subsequent larger studies will further demonstrate the therapeutic potential of nivolumab as a monotherapy in a second-line setting for patients who progress on sorafenib or other TKIs. Moreover, data from larger, multicenter, randomized, controlled, phase III clinical trials is warranted for further assessment of the survival benefits. Third, the lack of stratification and the presence of differences in baseline characteristics across treatment arms further confounded the interpretations of the study. Significant differences existed in terms of underlying co-morbidities, age, ECOG status, BCLC stage, extravascular invasion, and CTP class. While the lower age of the nivolumab cohort is generally in favor of a potential survival advantage, the other variables mostly favored the sorafenib-only arm. Despite these multiple disadvantages, nivolumab-treated patients fared better in terms of survival outcomes. Fourth, the two treatment cohorts were enrolled at different time points, and clear recruitment bias existed for the nivolumab arm, which represented a select group of TKI-unresponsive patients. Over the past decade, clinicians have become more adept at identifying suitable patients for systemic therapies and better handling their TRAEs, allowing longer exposure to these systemic therapies. This may partly account for the more favorable responses seen in nivolumab. Crucially, the sorafenib-only arm comprised patients who were both treatment-responsive as well as those with progressive disease. Historically, it was not uncommon for patients to remain on sorafenib despite disease progression due to a lack of treatment alternatives. The inclusion of only sorafenib-resistant patients in the sorafenib-only arm, as with nivolumab-treated patients, would have introduced a potential bias against the sorafenib-only arm. These confounders in the study design can best be addressed by a placebo-controlled, randomized study for second-line nivolumab therapy in TKI-unresponsive patients.

## 5. Conclusions

In conclusion, these early real-life data offer encouraging results for nivolumab as an effective second-line treatment option for patients with uHCC who experience disease progression while on sorafenib treatment, with significantly improved OS. These results advocate for the early uptake of systemic therapies, such as nivolumab, in compensated cirrhosis and patients with intermediate-stage HCC that are unsuitable for or unresponsive to transarterial chemoembolization. Further large, randomized, controlled studies or collaborations leveraging multicenter databases could provide a more diversified patient population, and validate the efficacy and safety of nivolumab as a monotherapy in a second-line setting, thereby enhancing the generalizability of these findings.

## Figures and Tables

**Table 1:** Baseline characteristics of patients in the sorafenib group and in the sorafenib followed by second-line nivolumab group.

Variable	Total (*n* = 80)	Sorafenib (*n* = 38)	Nivolumab (*n* = 42)	*p*
Age (yrs)	72.4 ± 10.1	75.0 ± 9.8	70.0 ± 9.8	0.024
Male gender	70 (87.5)	34 (89.5)	36 (85.7)	0.74
BMI (kg/m^2^)	25.4 ± 5.4	25.7 ± 6.0	25.2 ± 4.9	0.69
Comorbidities				
Diabetes	24 (30.0)	5 (13.2)	19 (45.2)	0.002
Hypertension	24 (30.0)	6 (15.8)	18 (42.9)	0.008
CKD	5 (6.3)	0 (0.0)	5 (11.9)	0.06
Cardiac disease	5 (6.3)	2 (5.3)	3 (7.1)	1.00
ECOG				
PS 0–1	65 (81.3)	32 (84.2)	33 (78.6)	0.52
PS 2–3	15 (18.8)	6 (15.8)	9 (21.4)	
AFP (ng/mL)	41.4 (6–1501)	28.9 (6.3–790.2)	50.6 (6.0–2670.0)	0.82
<400	52 (65.0)	26 (68.4)	26 (61.9)	0.54
≥400	28 (35.0)	12 (31.6)	16 (38.1)	
Etiology				
HBV	27 * (33.8)	16 * (42.1)	11 (26.2)	0.13
HCV	20 * (25.0)	10 * (26.3)	10 (23.8)	0.80
Non-viral	34 (42.5)	13 (34.2)	21 (50.0)	0.15
CTP class A/B	57 (71.3)/23 (28.8)	30 (78.9)/8 (21.1)	27 (64.3)/15 (35.7)	0.15
BCLC stage B/C	28 (35)/52 (65)	20 (52.6)/18 (47.4)	8 (19.0)/34 (81.0)	0.002
PV thrombosis	36 (45.0)	16 (42.1)	20 (47.6)	0.62
Metastases	31 (38.8)	8 (21.1)	23 (54.8)	0.002
Hepatic decompensation				
Variceal bleeding	9 (11.3)	4 (10.5)	5 (11.9)	1.00
Ascites	12 (15.0)	5 (13.2)	7 (16.7)	0.66
PSE	3 ** (3.8)	1 ** (2.6)	2 ** (4.8)	1.00
Creatinine	81.0 ± 33.7	76.7 ± 17.8	84.9 ± 43.2	0.28

Data presented as n (%), mean ± standard deviation, or median (interquartile range), as appropriate. * One patient with HBV-HCV co-infection. ** in association with variceal bleeding. BMI, body mass index; CTP, Child-Turcotte-Pugh; CKD, chronic kidney disease; ECOG, Eastern Cooperative Oncology Group; PS, performance status; AFP, alfa-fetoprotein; BCLC, Barcelona Clinic Liver Cancer; PV, portal vein.

**Table 2:** Baseline characteristics of CTP class A patients in the sorafenib group and in the sorafenib followed by second-line nivolumab group.

Variable	Total (*n* = 57)	Sorafenib (*n* = 30)	Nivolumab (*n* = 27)	*p*
Age (yrs)	74.9 ± 8.8	76.6 ± 9.8	73.0 ± 7.2	0.13
Male gender	51 (89.5)	27 (90.0)	24 (88.9)	0.89
Comorbidities				
Diabetes	14 (24.6)	4 (13.3)	10 (37.0)	0.038
Hypertension	16 (28.1)	11 (40.7)	5 (16.7)	0.043
CKD	3 (5.3)	0 (0)	3 (11.1)	0.06
Cardiac disease	3 (5.3)	2 (6.7)	1 (3.7)	0.62
ECOG				
PS 0–1	46 (80.7)	26 (86.7)	20 (74.1)	0.23
PS 2–3	11 (19.3)	4 (13.3)	7 (25.9)	
AFP (ng/mL)	55.5 (6.2–4058)	28.9 (6.8–688.2)	690.2 (6–16480)	0.99
<400	34 (59.6)	21 (70.0)	13 (48.1)	0.09
≥400	23 (40.4)	9 (30.0)	14 (51.9)	
Etiology				
HBV	20 * (35.1)	12 * (40.0)	8 (29.6)	0.41
HCV	11 * (19.3)	6 * (20.0)	5 (18.5)	0.89
Non-viral	27 (47.4)	13 (43.3)	14 (51.9)	0.52
BCLC stage B/C	20(35)/37(65)	15 (50)/15(50)	5 (18.5)/22 (81.5)	0.013
PV thrombosis	27 (47.7)	13 (43.3)	14 (51.9)	0.52
Metastases	24 (42.1)	7 (21.3)	17 (63.0)	0.002
Variceal bleeding	6 (10.5)	3 (11.1)	3 (10.0)	0.89
PSE	2 ** (3.5)	1 ** (3.7)	1 ** (3.3)	0.94
Creatinine	79.6 ± 27.4	76.2 ± 16.2	84.4 ± 36.1	0.33

Data presented as n (%), mean ± standard deviation, or median (interquartile range), as appropriate. * One patient with HBV-HCV co-infection. ** in association with variceal bleeding. CTP, Child-Turcotte-Pugh; CKD, chronic kidney disease; ECOG, Eastern Cooperative Oncology Group; PS, performance status; AFP, alfa-fetoprotein; BCLC, Barcelona Clinic Liver Cancer; PV, portal vein; PSE, portosystemic encephalopathy.

**Table 3:** Baseline characteristics of BCLC stage B patients in the sorafenib group and in the sorafenib followed by second-line nivolumab group.

Variable	Total (*n* = 28)	Sorafenib (*n* = 20)	Nivolumab (*n* = 8)	*p*
Age (yrs)	72.7 ± 9.0	74.6 ± 8.9	64.9 ± 8.9	0.07
Male gender	23 (82.1)	17 (85.0)	6 (75.0)	0.53
Comorbidities				
Diabetes	9 (32.1)	4 (20.0)	5 (62.5)	0.07
Hypertension	9 (32.1)	4 (20.0)	5 (62.5)	0.07
CKD	1 (3.6)	0 (0)	1 (12.5)	0.29
Cardiac disease	2 (7.1)	1 (5.0)	1 (12.5)	0.50
ECOG				
PS 0–1	26 (92.9)	18 (90.0)	8 (100)	1.00
PS 2–3	2 (7.1)	2 (10.0)	0 (0)	
AFP (ng/mL)	9.2 (6.0–611.7)	9.2 (6.0–848.7)	10.8 (6.0–42.5)	0.36
<400	21 (75.0)	14 (70.0)	7 (87.5)	0.63
≥400	7 (25.0)	6 (30.0)	1 (12.5)	
Etiology				
HBV	7 (25.0)	6 (30.0)	1 (12.5)	0.63
HCV	6 (21.4)	4 (20.0)	2 (25.0)	1.00
Non-viral	15 (53.6)	10 (50.0)	5 (62.5)	0.69
CTP class A/B	20 (71.4)/8 (28.6)	15 (75)/5 (25)	5 (62.5)/3 (37.5)	0.65
Hepatic decompensation				
Variceal bleeding	4 (14.3)	2 (10.0)	2 (25.0)	0.56
Ascites	3 (10.7)	3 (15.0)	0 (0)	0.54
PSE	0 (0)	-	-	-
Creatinine	81.7 ± 32.2	79.7 ± 19.1	86.8 ± 54.4	0.61

Data presented as *n* (%), mean ± standard deviation, or median (interquartile range), as appropriate. CTP, Child-Turcotte-Pugh; CKD, chronic kidney disease; ECOG, Eastern Cooperative Oncology Group; PS, performance status; AFP, alfa-fetoprotein; BCLC, Barcelona Clinic Liver Cancer; PSE, portosystemic encephalopathy.

**Table 4:** Tumor response in the overall study population based on treatment received according to Response Evaluation Criteria in Solid Tumors (RECIST1.1).

Tumor Response	Sorafenib (*n* = 38)	Nivolumab (*n* = 42)	*p*
Stable disease	13 (34.2)	10 (23.8)	0.409
Partial response	3 (7.9)	1 (2.4)	
Progressive disease	14 (36.8)	18 (42.9)	
Not evaluable	2 (5.3)	3 (7.1)	
Missing	6 (15.8)	10 (23.8)	

Data presented as *n* (%).

## Data Availability

The data that support the findings of this study are available on request from the corresponding author [FMS]. The data are not publicly available due to [restrictions arising from containing information that could compromise the privacy of research participants]. Release of raw data would require express approval from institutional review boards governing the conduct of this study.

## Supplementary Materials

The following supporting information can be downloaded at: https://www.mdpi.com/article/10.3390/cancers16122196/s1, Supplementary Figure S1: Study schematic showing patient disposition in both treatment groups. Supplementary Figure S2: Time from the start of sorafenib to progression on sorafenib in patients receiving sorafenib only or sorafenib followed by second-line nivolumab treatment. Supplementary Figure S3: Survival analysis of the overall cohort of patients on the basis of (A) CTP class and (B) BCLC stage. Supplementary Table S1. Summary of safety events (>5%) among patients treated with sorafenib and nivolumab.

## Abbreviations

HBV, hepatitis B virus; HCV, hepatitis C virus; ALT, alanine aminotransferase; AST, aspartate aminotransferase; ALP, alkaline phosphatase; AFP, α-fetoprotein; HBsAg, hepatitis B surface antigen; ULN, upper limit of normal; BMI, body mass index; HCC, hepatocellular carcinoma; BCLC, Barcelona Clinic Liver Cancer; ECOG, Eastern Cooperative Oncology Group.
