# Supplementary material for: Nivolumab as Second-Line Therapy Improves Survival in Patients with Unresectable Hepatocellular Carcinoma
Source: Cancers (Basel). 2024 Jun 11;16(12):2196. doi: 10.3390/cancers16122196 (PMC11202187; doi:10.3390/cancers16122196)
Supplement: Supplementary file 1 [file cancers-16-02196-s001.zip › cancers-2941108-supplementary figures.pdf]

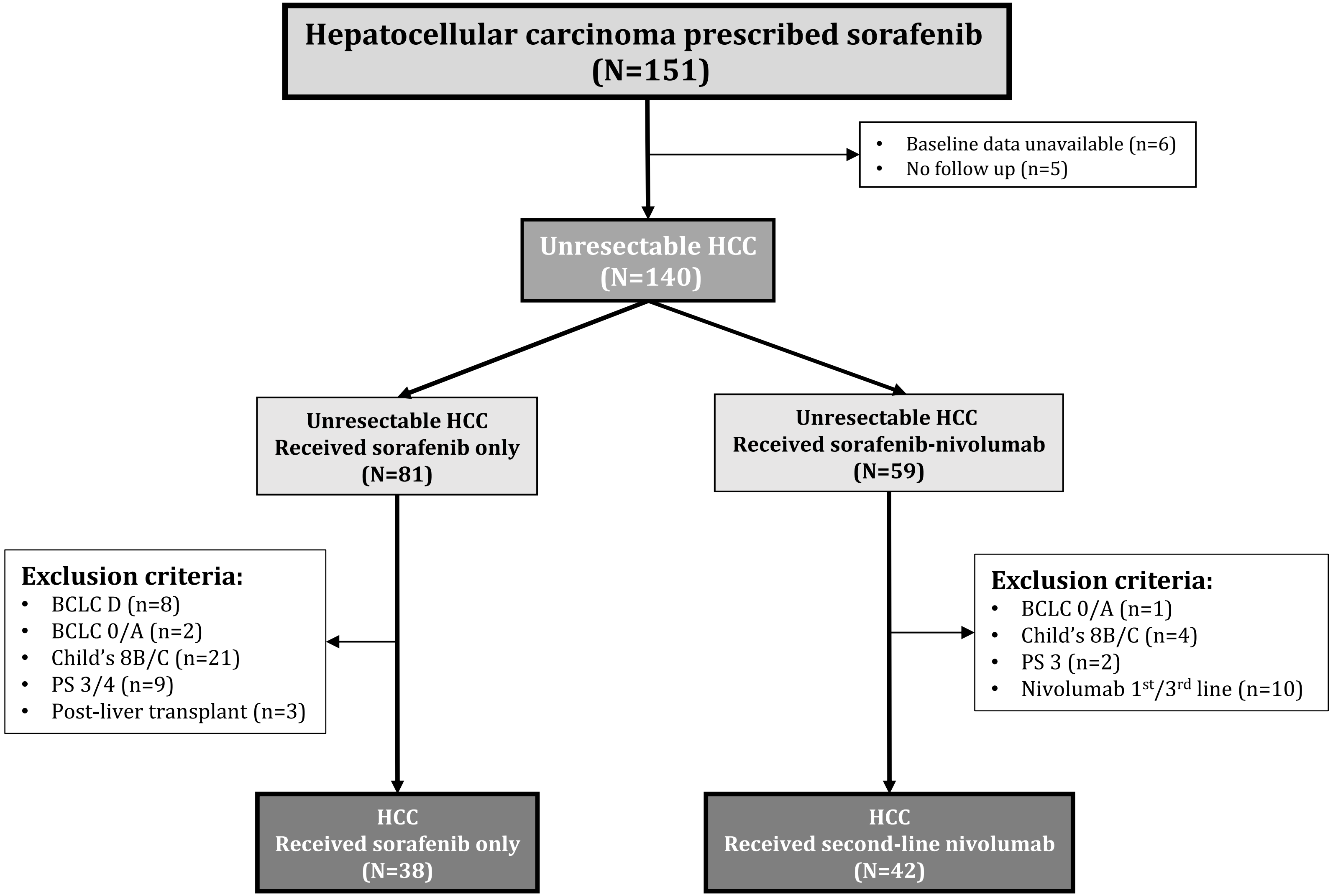

Supplementary Figure S1: Study schematic showing patient disposition in both treatment groups

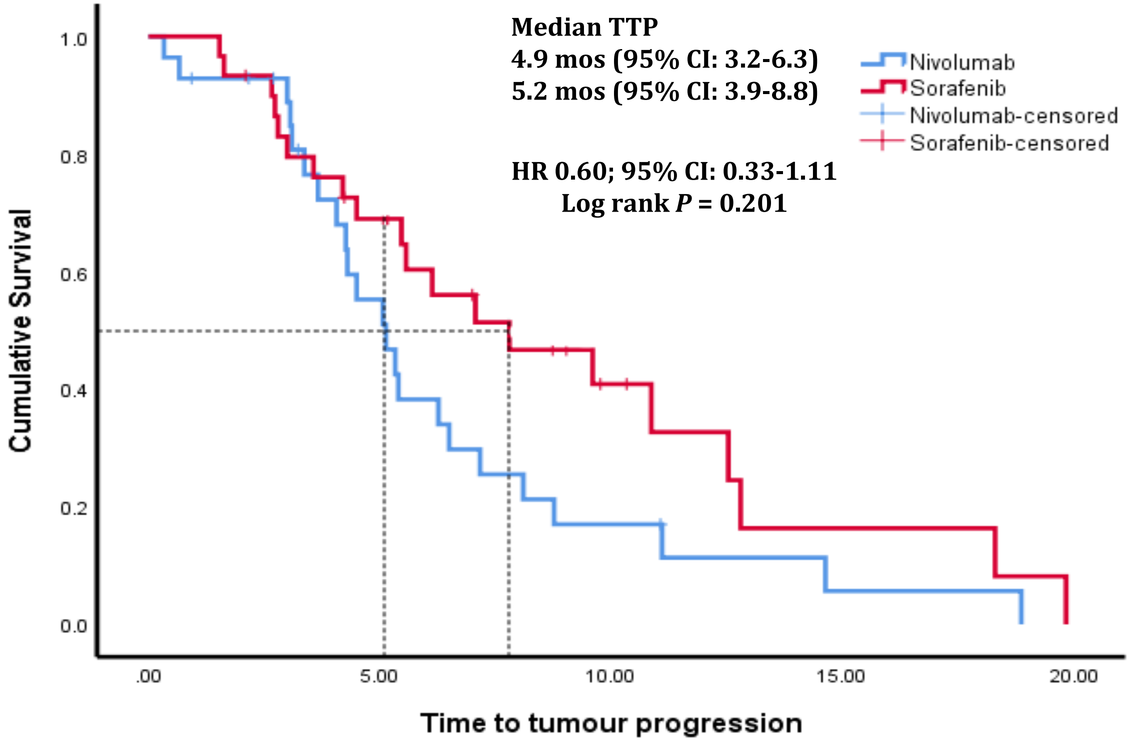

Supplementary Figure S2: Time from the start of sorafenib to progression on sorafenib in patients receiving sorafenib only, or sorafenib followed by second-line nivolumab treatment

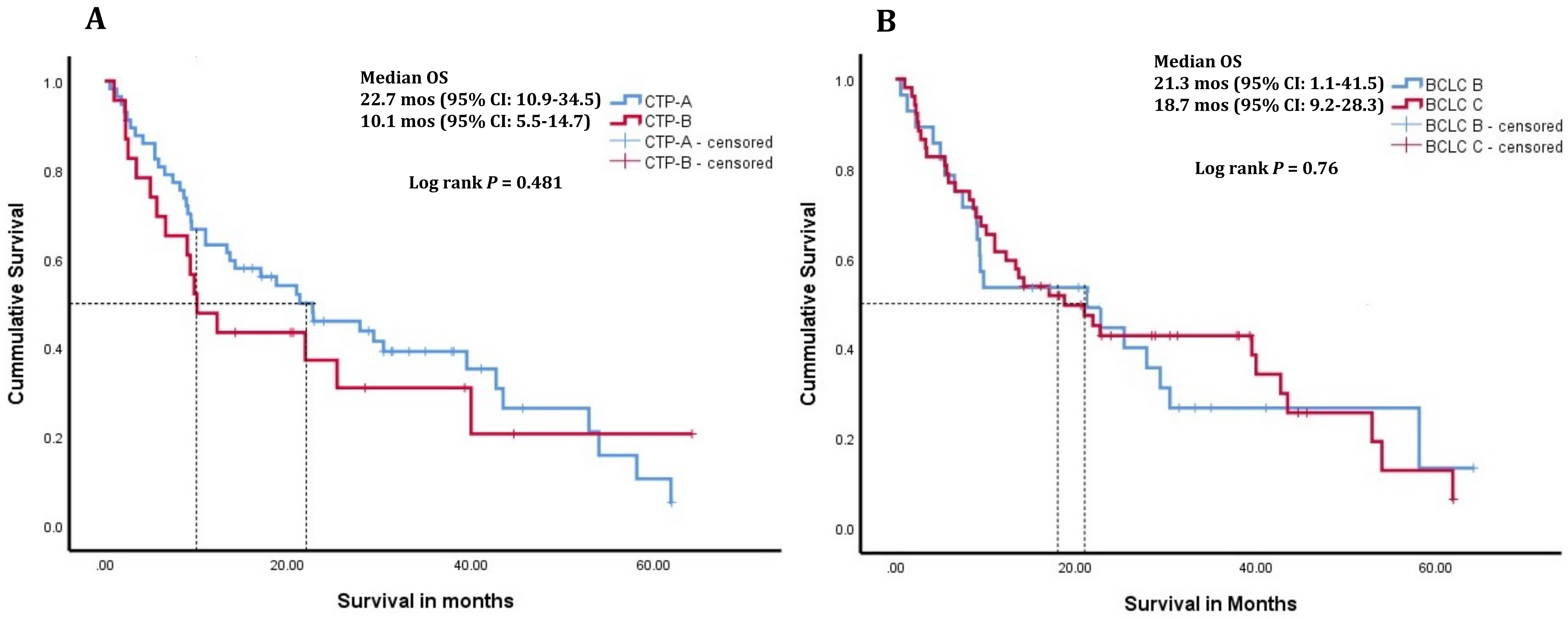

Supplementary Figure S3: Survival analysis of overall cohort of patients on the basis of A) CTP class and by B) BCLC stage
