# Supplementary material for: Nivolumab as Second-Line Therapy Improves Survival in Patients with Unresectable Hepatocellular Carcinoma
Source: Cancers (Basel). 2024 Jun 11;16(12):2196. doi: 10.3390/cancers16122196 (PMC11202187; doi:10.3390/cancers16122196)
Supplement: Supplementary file 1 [file cancers-16-02196-s001.zip › cancers-2941108-supplementary table.pdf]

Supplementary Table S1. Summary of safety events (>5%) among patients treated with sorafenib and nivolumab.

Adverse event	Sorafenib only (N=38)	Nivolumab (N=42)
Transaminitis	2 (5.3)	3 (7.1)
Hyperbilirubinemia	2 (5.3)	2 (4.8)
Pruritus	4 (10.5)	2 (4.8)
Skin rash	7 (18.4)	4 (9.5)
Gastrointestinal	6 (15.8)	(4.8)
SAEs or death	0 (0)	0 (0)

Data presented as n (%). SAE, serious adverse events.
